# Association of different inflammatory indices with risk of early natural menopause: a cross-sectional analysis of the NHANES 2013–2018

**DOI:** 10.3389/fmed.2024.1490194

**Published:** 2024-11-28

**Authors:** Mengyu Zheng, Junying Li, Yushan Cao, Zhuo Bao, Xing Dong, Pei Zhang, Jinxiang Yan, Yixuan Liu, Yongzhen Guo, Xianxu Zeng

**Affiliations:** ^1^Department of Gynecology and Obstetrics, The Third Affiliated Hospital of Zhengzhou University, Zhengzhou, China; ^2^Zhengzhou Key Laboratory of Gynecological Disease’s Early Diagnosis, Zhengzhou, China; ^3^Department of Pathology, The Third Affiliated Hospital of Zhengzhou University, Zhengzhou, China

**Keywords:** early natural menopause, inflammatory indices, peripheral blood cell counts, cross-sectional study, NHANES

## Abstract

**Background:**

Early natural menopause, characterized by the cessation of ovarian function before the age of 45, has been a subject of prior research indicating that inflammation may predict the onset of menopause. However, the specific relationship between peripheral blood inflammatory parameters and early natural menopause remains ambiguous.

**Methods:**

This observational study utilized data from the National Health and Nutrition Examination Survey (NHANES) spanning the years 2013–2018. The age at menopause was ascertained through the Reproductive Health Questionnaire (RHQ), with early natural menopause defined as menopause occurring before the age of 45 years. Complete blood counts were derived from laboratory test data, and seven indices of inflammation were calculated, including lymphocyte count (LC), neutrophil count (NC), systemic immune inflammation index (SII), product of platelet and neutrophil count (PPN), platelet-lymphocyte ratio (PLR), neutrophil-lymphocyte ratio (NLR), and lymphocyte-monocyte ratio (LMR). A multivariate weighted logistic regression analysis was employed to estimate the association between these inflammatory indices and early natural menopause.

**Results:**

A total of 2,034 participants were included in the analysis, of whom 460 reported experiencing menopause before the age of 45. Both Log2-NC and Log2-PPN were found to be positively correlated with early menopause, with odds ratios (OR) of 1.56 (95% CI: 1.16, 2.09; *p* = 0.005) and 1.36 (95% CI: 1.07, 1.72; *p* = 0.015), respectively. The results from models 1 and 2 were consistent with those from model 3. In the trend test, participants in the fourth quartile (Q4) of log2-LC exhibited a positive correlation with early menopause compared to those in the lowest quartile (Q1), with an OR of 1.41 (95% CI: 1.03, 1.93; *p* = 0.033). Similarly, the fourth quartile (Q4) of log2-NC and log2-PPN demonstrated a positive correlation with early menopause, with odds ratios (OR) of 1.76 (95% CI: 1.27–2.45; *p* < 0.001) and 1.66 (95% CI: 1.21–2.29; *p* = 0.002), respectively. In Model 3, log2-SII, log2-PLR, log2-NLR, and log2-LMR were not significantly associated with early menopause.

**Conclusion:**

Our findings indicate that elevated levels of Log2-LC, Log2-NC, and Log2-PPN are positively correlated with an increased risk of early menopause among women in the United States.

## 1 Introduction

In Western populations, the prevalence of early natural menopause exceeds 10% (1). Current research indicates that women who experience early menopause face an elevated risk of overall mortality, cardiovascular disease, neurological disorders, psychiatric conditions, osteoporosis, and other sequelae (2), which will inevitably augment the future public health burden. Furthermore, as an increasing number of women are postponing childbearing, the implications of early menopause on family planning are substantial. Consequently, early identification of individuals at risk for early menopause may facilitate the prevention of these adverse outcomes.

Inflammation represents a prevalent pathophysiological process, characterized as a local and systemic defense response to both exogenous and endogenous injury factors. Numerous immune cells play critical roles in physiological events such as follicular development, ovulation, luteal formation, and atrophy. During these processes, a substantial array of inflammatory chemokines, pro-inflammatory factors, stromal proteases, prostaglandins, and plasminogen activators are synthesized and secreted, contributing to regulatory mechanisms (3). Menopause, indicative of ovarian aging, also involves the participation of immune factors in its regulatory pathways. Autoimmune abnormalities are responsible for 10–30% of cases of early-onset ovarian dysfunction, predominantly characterized by the presence of anti-ovarian autoantibodies and immune-mediated oophoritis (4).

Peripheral blood components, including white blood cells, neutrophils, lymphocytes, and platelets, play a critical role in the inflammatory response. These parameters can be readily obtained through routine blood examinations. Despite their limited specificity, these indices offer advantages such as low cost, repeatability, minimal invasiveness, and broad acceptance. An elevated count of neutrophils and platelets is commonly interpreted as an indicator of systemic inflammation. LC, NC, SII, PPN, PLR, NLR, and LMR are emerging inflammatory indices derived from peripheral blood cell counts that are used to predict disease prognosis. Recent studies have demonstrated that specific populations of immune cells, particularly lymphocytes, tend to accumulate in aging ovaries (5). SII represents a novel and stable inflammatory biomarker, calculated as the product of platelet count and neutrophil count divided by lymphocyte count. SII is utilized to assess both local and systemic inflammation, as well as the systemic immune response (6, 7). Emerging evidence indicates that SII plays a significant role in the onset, development, and progression of various cancers (8, 9), including cervical cancer (10), metastatic urothelial carcinoma (11), esophageal cancer (12), and hepatocellular carcinoma (13, 14). PPN was calculated by multiplying the peripheral blood platelet count by the neutrophil count, and it has been reported that PPN is positively correlated with female bone mineral density (15, 16). PLR is the ratio of platelets to lymphocytes, while NLR is the ratio of neutrophils to lymphocytes. Both PLR and NLR have been found to correlate with prognosis in breast cancer. Specifically, a high NLR is associated with a poor prognosis in breast cancer, and an elevated PLR may also be indicative of a poor prognosis in breast cancer patients (17). Furthermore, current findings suggest that the PLR and NLR are essential parameters for predicting prognosis in patients with stage IIB-III cervical cancer receiving radiotherapy (18). The LMR has recently been employed to assess the survival value in various solid cancers (19–21). The researchers additionally identified positive correlations between SII, NLR, PPN, and NC with female estradiol levels. A cross-sectional study has demonstrated that SII and PLR are negatively correlated with female infertility (22). However, to the best of our knowledge, no studies have investigated the relationship between various peripheral blood inflammatory indices and early menopause in women.

Therefore, the objective of this study was to investigate the potential association between various peripheral blood inflammation indices and the onset of early menopause in women. This analysis utilized a nationally representative sample of American women derived from the NHANES, aiming to contribute novel insights into the management of women’s health.

## 2 Materials and methods

### 2.1 Data source and population selection

**Figure 1:**
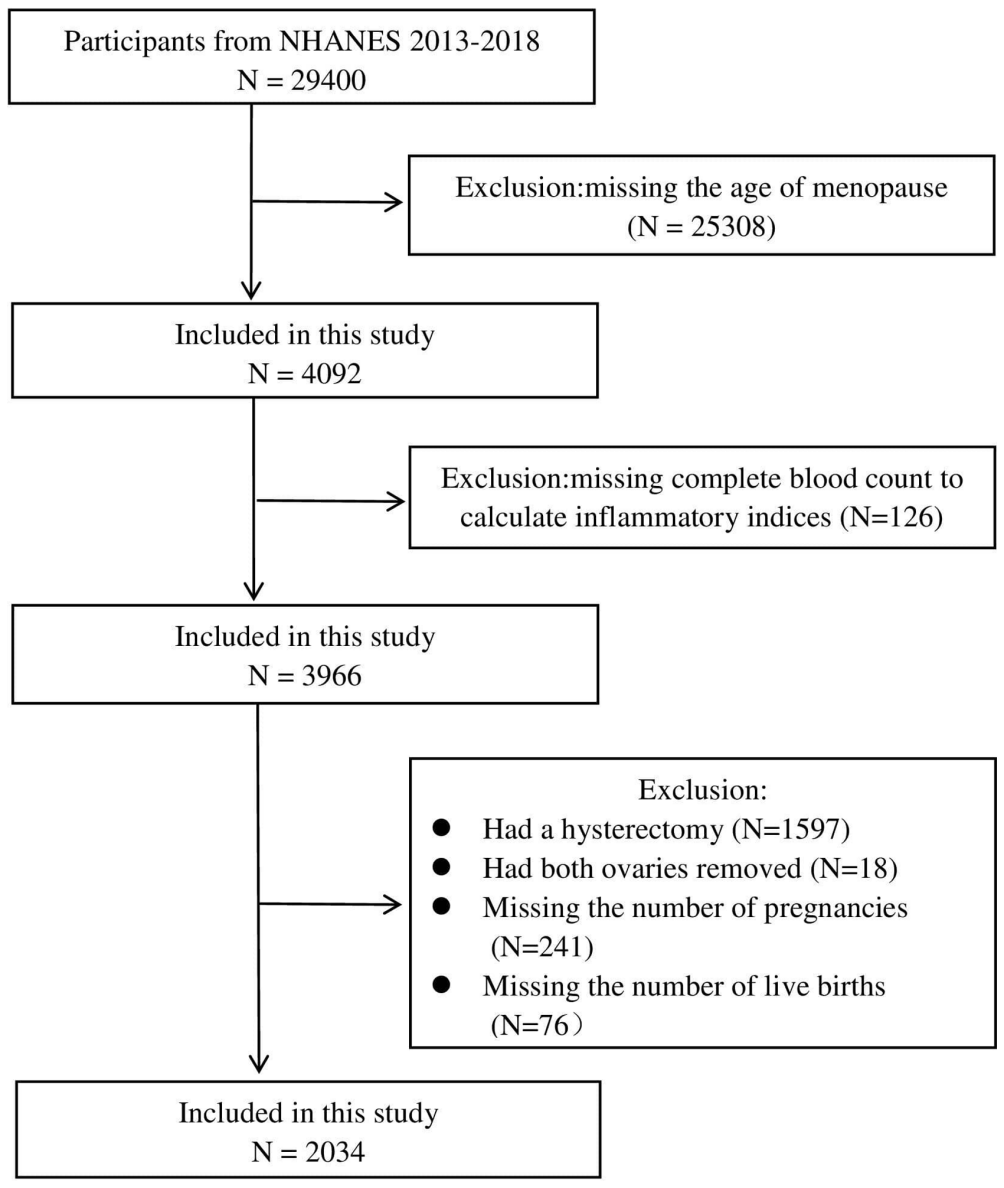
Flowchart of the research populations: NHANES 2013–2018.

To furnish comprehensive data and address pivotal public health concerns impacting the United States population, the National Center for Health Statistics (NCHS) developed and implemented the National Health and Nutrition Examination Survey (NHANES). This extensive, nationally representative, cross-sectional survey is conducted biennially through both questionnaires and physical examinations. For our analysis, we extracted data from 2,034 female participants from the NHANES 2013–2018 database (Figure 1). Participants in each NHANES cycle were selected using a stratified, multistage probability sampling method. The Research Ethics Review Board of the NCHS granted approval for the NHANES study, and informed written consent was obtained from all participants.

### 2.2 Definition of early menopause and inflammatory indices

We obtained lymphocyte count (LC), platelet count (PC), neutrophil count (NC), and monocyte count (MC), expressed in units of 1,000 cells/μL, from complete blood count inflammatory indices analyses. These data were utilized to calculate various inflammatory indices as follows: SII was determined using the formula PC*NC/LC; PPN was calculated as the product of platelet count and neutrophil count; PLR was defined as the ratio of platelet count to lymphocyte count; NLR was defined as the ratio of neutrophil count to lymphocyte count; and LMR was defined as the ratio of lymphocyte count to monocyte count.

Self-reported menopause was assessed based on responses to question RHQ060 from the Reproductive Health Questionnaire, which inquires about the “Age at last menstrual period.” Menopause was defined as occurring after 12 consecutive months of amenorrhea. The study sample comprised 2034 women with no history of hysterectomy or oophorectomy. Early menopause was classified as natural menopause occurring before the age of 45 years.

### 2.3 Covariates

Based on clinical practice (23–29), previous literature, and data available in the NHANES database, we selected the following covariates to control for potential confounding bias in this study: race, marital status, education level, income-to -poverty ratio (PIR), body mass index (BMI), smoking status, alcohol use, infertility, pelvic inflammatory disease (PID), age at menarche, number of pregnancies, number of live births, and history of diabetes, hypertension, high cholesterol levels, and cancer. BMI was stratified into three categories based on clinical significance: normal (<25 kg/m^2^), overweight (25–30 kg/m^2^), and obesity (≥30 kg/m^2^). Racial classification included the following groups: “Mexican American,” “Other Hispanic,” “Non-Hispanic White,” “Non-Hispanic Black,” and “Other/more than one race.” Marital status was dichotomized into “Married” and “Unmarried or other.” Educational attainment was categorized into three levels: “Less than 11th grade,” “High school or GED,” and “Some college or AA degree and above.” Smoking status was classified as either “Yes” or “No” based on self-reported consumption of at least 100 cigarettes over the individual’s lifetime. Alcohol use was similarly categorized as “Yes” or “No” based on self-reported consumption of at least 12 alcoholic drinks per year. The PIR was stratified into three categories: less than 1.5, between 1.5 and 3.5, and greater than or equal to 3.5. Self-reported infertility was assessed through responses to two specific items (questions RHQ074 and RHQ076) from the Reproductive Health Questionnaire. Question RHQ074 inquired, “Have you ever attempted to become pregnant over a period of at least a year without success?” Similarly, Question RHQ076 asked, “Have you ever consulted a doctor or other medical provider due to an inability to conceive?” Women who responded affirmatively to either of these questions were classified as having a history of infertility. Those who answered “Yes” were subsequently categorized into the “non-infertility group.” The presence of PID was determined based on responses to Question RHQ078 from the Reproductive Health Questionnaire, which asked, “Have you ever been treated for a pelvic infection/PID?”

### 2.4 Statistical analysis

The weight utilized for analysis was selected in accordance with the guidelines provided by the NHANES database, which recommend the application of the mobile examination center exam weight (WTMEC2YR) due to the complete blood count being measured in the mobile examination center. Given that the distributions of LC, NC, SII, PPN, PLR, NLR, and LMR among the individuals included in the present study were right-skewed, these variables were log2-transformed prior to data analysis (Supplementary material).

In our study, continuous variables were reported as means and standard deviations, while categorical variables were expressed as frequencies (*n*) and percentages (%). Participants were stratified into two groups based on the age at menopause: “age of menopause ≥45” and “age of menopause <45.” The differences between the groups were analyzed using the Chi-square test for categorical variables and the Wilcoxon test for continuous variables. We employed a survey-weighted multivariable logistic regression model to evaluate the association between various inflammatory indices and the incidence of early natural menopause, quantifying the relationship using odds ratios (OR) and 95% confidence intervals (95% CI). In the analysis, we developed three models: Model I without any adjustments; Model II adjusted for race, marital status, education level, PIR, BMI, smoking status, alcohol use; and Model III adjusted for race, marital status, education level, PIR, BMI, smoking status, alcohol use, and histories of diabetes, hypertension, hypercholesterolemia and PID, cancer, number of pregnancies, number of live births, age at menarche. Based on the outcomes of these analyses, we conducted a further evaluation of the differences in the risk of early natural menopause among the quartile groups of various inflammatory indices, using the Q1 group as the reference. Additionally, we employed restricted cubic spline (RCS) curves derived from Model III to investigate potential non-linear relationships between the inflammatory indices and early natural menopause. Furthermore, we conducted interaction and stratified analyses based on race, marital status, education level, PIR, BMI, smoking status, alcohol use, and histories of diabetes, hypertension, high cholesterol levels, and PID. The statistical software utilized for data analysis included R (version 4.3.1, http://www.Rproject.org) and EmpowerStats (www.empowerstats.com; X&Y Solutions, Inc., Boston, MA). A *p* value of less than 0.05 was considered statistically significant.

## 3 Results

### 3.1 Baseline characteristics

A total of 2,034 individuals were included in the analysis, of whom 460 reported experiencing menopause before the age of 45. Baseline characteristics of the included women, derived from the NHANES 2013 to 2018 data, are presented in Tables 1, 2. Compared to women who did not experience early menopause, those who did were more likely to be of Mexican American or Non-Hispanic White and Non-Hispanic Black ethnicity, possess lower educational attainment, have a lower PIR, and lack histories of hypertension and high cholesterol levels (*p* < 0.05). No significant differences were observed between the two groups with respect to BMI, alcohol use, number of pregnancies, and number of live births. The mean log2-transformed values for LC, NC, PPN, and LMR in women experiencing early menopause were 1.13 (0.52) * 1,000 cells/μL, 2.00 (0.58) * 1,000 cells/μL, 9.90 (0.78) * 1,000 cells/μL, and 2.06 (0.56) * 1,000 cells/μL, respectively, which were significantly higher compared to those in women not experiencing early menopause. Conversely, the mean log2-transformed PLR in women with early menopause was 6.77 (0.56) * 1000 cells/μL, significantly lower than that in women without early menopause. No significant differences were observed in log2-SII and log2-NLR between women experiencing early menopause and those not experiencing early menopause.

**Table 1:** Baseline characteristics of included women from NHANES 2013 to 2018.

Variables	Age of menopause ≥ 45 (*n* = 1,574)	Age of menopause < 45 (*n* = 460)	*p*
Age (mean ± SD)	64.10 (8.98)	54.93 (15.91)	<0.001*
Number of pregnancies (mean ± SD)	3.74 (2.04)	3.64 (2.09)	0.386
Number of live births (mean ± SD)	2.96 (1.77)	2.83 (1.78)	0.172
Age at menarche (mean ± SD)	19.84 (82.19)	23.48 (102.50)	0.432
Race (%)			0.005*
Mexican American	241 (15.3)	87 (18.9)	
Other Hispanic	209 (13.3)	49 (10.7)	
Non-Hispanic White	600 (38.1)	186 (40.4)	
Non-Hispanic Black	278 (17.7)	93 (20.2)	
Other/more than one race	246 (15.6)	45 (9.8)	
Marital status (%)			0.006*
Married	791 (50.3)	197 (42.8)	
Unmarried or other	783 (49.7)	263 (57.2)	
Education level (%)			0.024*
Less than 11th grade	410 (26.0)	132 (28.7)	
High school or GED	358 (22.7)	125 (27.2)	
Some college or AA degree above	806 (51.2)	203 (44.1)	
Income-to-poverty ratio (PIR) (%)			<0.001*
0–1.3RIP	429 (27.3)	165 (35.9)	
>1.3–3.5 RIP	533 (33.9)	153 (33.3)	
>3.5 RIP	612 (38.9)	142 (30.9)	
Body mass index (BMI) (%)			0.786
Normal or low weight	419 (26.6)	122 (26.5)	
Overweight	465 (29.5)	129 (28.0)	
Obesity	690 (43.8)	209 (45.4)	
Smoking status (%)			0.013*
No	555 (35.3)	192 (41.7)	
Yes	1,019 (64.7)	268 (58.3)	
Alcohol use (%)			0.711
No	592 (37.6)	168 (36.5)	
Yes	982 (62.4)	292 (63.5)	
Diabetes (%)			0.131
No	1,209 (76.8)	373 (81.1)	
Yes	315 (20.0)	73 (15.9)	
Borderline	50 (3.2)	14 (3.0)	
Hypertension (%)			<0.001*
No	723 (45.9)	266 (57.8)	
Yes	851 (54.1)	194 (42.2)	
High cholesterol levels (%)			<0.001*
No	808 (51.3)	289 (62.8)	
Yes	766 (48.7)	171 (37.2)	
Infertility (%)			<0.001*
No	488 (31.0)	231 (50.2)	
Yes	45 (2.9)	28 (6.1)	
Missing	1,041 (66.1)	201 (43.7)	
Pelvic inflammatory disease (PID) (%)			<0.001*
No	496 (31.5)	235 (51.1)	
Yes	34 (2.2)	22 (4.8)	
Missing	1,044 (66.3)	203 (44.1)	
Cancer (%)			0.998
No	1,363 (86.6)	399 (86.7)	
Yes	211 (13.4)	61 (13.3)	

*mean *p* < 0.05.

**Table 2:** Baseline characteristics of included women from NHANES 2013 to 2018.

Inflammatory indexes	Age of menopause ≥ 45	Age of menopause < 45	*p*
*N*	1,574	460	
Log2-LC (mean ± SD)	1.04 (0.53)	1.13 (0.52)	0.001*
Log2-PC (mean ± SD)	7.87 (0.35)	7.90 (0.38)	0.104
Log2-NC (mean ± SD)	1.90 (0.55)	2.00 (0.58)	0.001*
Log2-MC (mean ± SD)	−0.95 (0.48)	−0.93 (0.49)	0.567
Log2-SII (mean ± SD)	8.73 (0.77)	8.77 (0.80)	0.317
Log2-PPN (mean ± SD)	9.77 (0.74)	9.90 (0.78)	0.001*
Log2-PLR (mean ± SD)	6.83 (0.54)	6.77 (0.56)	0.042*
Log2-NLR (mean ± SD)	0.86 (0.68)	0.87 (0.68)	0.780
Log2-LMR (mean ± SD)	1.99 (0.57)	2.06 (0.56)	0.012*

If it is a continuous variable, it was obtained by Kruskal Wallis rank sum test. For those continuous variables having a theoretical number < 10, Fisher’s exact probability test was employed to calculate the *p* value. In the case of categorical data, the *p* value was calculated using weighted chi-square. *mean *p* < 0.05.

### 3.2 The associations between inflammatory indices and early natural menopause

The associations between inflammatory indices and early natural menopause are presented in Table 3. In Model 1, the log2-transformed neutrophil count (log2-NC) was positively associated with early natural menopause, with an odds ratio (OR) of 1.56 (95% confidence interval [CI]: 1.16, 2.09; *p* = 0.005). This association remained consistent in Model 2 and Model 3, with ORs of 1.54 (95% CI: 1.12, 2.21; *p* = 0.009) and 1.65 (95% CI: 1.21, 2.25; *p* = 0.005), respectively. The findings were consistent in Log2-PPN. In model 1, Log2-PLR exhibited a negative association with early natural menopause (OR 0.72; 95% CI: 0.56, 0.93; *p* = 0.016), which persisted in model 2 (OR 0.75; 95% CI: 0.60, 0.94; *p* = 0.015). However, this association was not statistically robust in model 3 (OR 0.87; 95% CI: 0.68, 1.12; *p* = 0.300). Conversely, Log2-LMR was positively associated with early natural menopause in both model 1 (OR 1.38; 95% CI: 1.12, 1.71; *p* = 0.004) and model 2 (OR 1.40; 95% CI: 1.12, 1.75; *p* = 0.006). However, the results were not significantly stable in Model 3 (OR 1.00; 95% CI: 0.75, 1.33; *p* = 0.993). Comparable findings were observed in the Log2-LC analysis. No significant association was identified between log2-SII, log2-NLR, and early natural menopause in Model 1, with ORs of 1.07 (95% CI: 0.88, 1.31; *p* = 0.493) and 1.01 (95% CI: 0.83, 1.23; *p* = 0.928), respectively. These results were consistent across Model 2 and Model 3.

**Table 3:** Associations between inflammatory indices and early menopsuse.

Inflammatory indices	Model 1			Model 2			Model 3		
	OR	(95% CI)	*p* value	OR	(95% CI)	*p* value	OR	(95% CI)	*p* value
Log2-LC	1.57	(1.22 ~ 2.03)	0.001*	1.52	(1.21 ~ 1.90)	0.001*	1.25	(0.97 ~ 1.60)	0.100
Log2-NC	1.56	(1.16 ~ 2.09)	0.005*	1.54	(1.12 ~ 2.11)	0.009*	1.65	(1.21 ~ 2.25)	0.005*
Log2-SII	1.07	(0.88 ~ 1.31)	0.493	1.06	(0.88 ~ 1.29)	0.542	1.20	(0.98 ~ 1.46)	0.098
Log2-PPN	1.36	(1.07 ~ 1.72)	0.015*	1.33	(1.05 ~ 1.69)	0.025*	1.35	(1.06 ~ 1.71)	0.024*
Log2-PLR	0.72	(0.56 ~ 0.93)	0.016*	0.75	(0.60 ~ 0.94)	0.015*	0.87	(0.68 ~ 1.12)	0.300
Log2-NLR	1.01	(0.83 ~ 1.23)	0.928	1.00	(0.83 ~ 1.21)	0.990	1.21	(0.96 ~ 1.52)	0.094
Log2-LMR	1.38	(1.12 ~ 1.71)	0.004*	1.40	(1.12 ~ 1.75)	0.006*	1.00	(0.75 ~ 1.33)	0.993

*mean *p* < 0.05.

Model 1: Non-adjusted.

Model 2: Adjusted for race, marital status, education level, Income -to -poverty ratio (PIR), Body mass index (BMI), smoking status, and alcohol use.

Model 3: Adjusted for model 2 + infertility, pelvic inflammatory disease (PID), number of pregnancies, number of live births, age at menarche, history of diabetes, hypertension, high cholesterol levels, and cancer.

Furthermore, when these inflammatory indices were converted from continuous to categorical variables by quartiles (Table 4), we observed that women in the highest log2-LC quartile group (Q4) exhibited a significantly increased risk of early natural menopause compared to those in the lowest log2-LC quartile group (Q1). This association was consistent across model 1 (OR 1.60; 95% CI: 1.19, 2.14; *p* = 0.002), model 2 (OR 1.62; 95% CI: 1.19, 2.19; *p* = 0.002), and model 3 (OR 1.41; 95% CI: 1.03, 1.93; *p* = 0.033). Similar associations were observed between log2-NC, log2-PPN quartiles and early natural menopause. Compared to the lowest quartile (Q1), the highest quartile (Q4) of log2-LMR exhibited a positive correlation with early natural menopause, with an odds ratio (OR) of 1.49 (95% confidence interval [CI]: 1.11, 2.00; *p* = 0.009) in model 1 and 1.64 (95% CI: 1.20, 2.25; *p* = 0.002) in model 2. However, in model 3, the highest quartile (Q4) of log2-LMR did not show a significant positive correlation with early natural menopause, with an OR of 1.27 (95% CI: 0.92, 1.77; *p* = 0.150). Additionally, no significant association was observed between quartiles of log2-SII, log2-PLR, or log2-NLR and early natural menopause in model 3.

**Table 4:** Associations between inflammatory indices and early menopsuse.

Inflammatory indices	Model 1		Model 2		Model 3	
	OR (95%CI)	*p*	OR (95%CI)	*p*	OR (95%CI)	*p*
Log2-LC						
Q1 [−1.32, 0.68]	Reference		Reference		Reference	
Q2 (0.77, 1.00]	1.09 (0.80 ~ 1.50)	0.581	1.12 (0.81 ~ 1.54)	0.497	1.10 (0.79 ~ 1.54)	0.566
Q3 (1.07, 1.32]	1.29 (0.94 ~ 1.76)	0.114	1.29 (0.94 ~ 1.78)	0.112	1.20 (0.86 ~ 1.66)	0.288
Q4 (1.38, 5.61]	1.60 (1.19 ~ 2.14)	0.002*	1.62 (1.19 ~ 2.19)	0.002*	1.41 (1.03 ~ 1.93)	0.033*
*p* for trend	<0.001*		0.001*		0.026*	
Log2-NC						
Q1 [−1.74, 1.54]	Reference		Reference		Reference	
Q2 (1.58, 1.93]	1.14 (0.84 ~ 1.55)	0.412	1.10 (0.81 ~ 1.51)	0.539	1.16 (0.84 ~ 1.61)	0.372
Q3 (1.96, 2.26]	1.24 (0.91 ~ 1.69)	0.176	1.19 (0.86 ~ 1.64)	0.292	1.23 (0.89 ~ 1.72)	0.214
Q4 (2.29, 3.94]	1.69 (1.26 ~ 2.28)	<0.001*	1.62 (1.18 ~ 2.22)	0.003*	1.76 (1.27 ~ 2.45)	<0.001*
*p* for trend	<0.001*		0.003*		<0.001*	
Log2-SII						
Q1 [4.29, 8.25]	Reference		Reference		Reference	
Q2 (8.26, 8.73]	1.04 (0.77 ~ 1.40)	0.807	1.02 (0.75 ~ 1.38)	0.913	1.02 (0.74 ~ 1.39)	0.918
Q3 (8.74, 9.20]	0.99 (0.74 ~ 1.34)	0.960	0.95 (0.70 ~ 1.29)	0.725	1.00 (0.73 ~ 1.37)	1.000
Q4 (9.21, 12.38]	1.16 (0.87 ~ 1.55)	0.325	1.11 (0.82 ~ 1.50)	0.518	1.15 (0.84 ~ 1.58)	0.373
*p* for trend	0.368		0.588		0.394	
Log2-PPN						
Q1 [5.04, 9.33]	Reference		Reference		Reference	
Q2 (9.34, 9.80]	1.10 (0.80 ~ 1.50)	0.565	1.06 (0.77 ~ 1.46)	0.703	1.16 (0.84 ~ 1.61)	0.367
Q3 (9.81, 10.26]	1.29 (0.95 ~ 1.75)	0.103	1.26 (0.92 ~ 1.72)	0.144	1.25 (0.90 ~ 1.72)	0.181
Q4 (10.27, 12.85]	1.69 (1.26 ~ 2.27)	<0.001*	1.61 (1.19 ~ 2.20)	0.002*	1.66 (1.21 ~ 2.29)	0.002*
*p* for trend	<0.001*		0.001*		0.001*	
Log2-PLR						
Q1 [1.99, 6.46]	Reference		Reference		Reference	
Q2 (6.47, 6.80]	0.61 (0.45 ~ 0.82)	0.001*	0.63 (0.46 ~ 0.85)	0.003*	0.62 (0.45 ~ 0.85)	0.003*
Q3 (6.81, 7.14]	0.77 (0.58 ~ 1.02)	0.068	0.76 (0.57 ~ 1.01)	0.057	0.78 (0.58 ~ 1.06)	0.110
Q4 (7.15, 9.43]	0.71 (0.53 ~ 0.94)	0.018*	0.72 (0.53 ~ 0.96)	0.028*	0.76 (0.56 ~ 1.04)	0.088
*p* for trend	0.046*		0.054		0.161	
Log2-NLR						
Q1 [−3.32, 0.44]	Reference		Reference		Reference	
Q2 (0.45, 0.85]	0.86 (0.64 ~ 1.16)	0.338	0.86 (0.63 ~ 1.17)	0.349	0.91 (0.67 ~ 1.25)	0.572
Q3 (0.86, 1.25]	1.02 (0.76 ~ 1.36)	0.917	0.99 (0.73 ~ 1.35)	0.974	1.09 (0.80 ~ 1.50)	0.585
Q4 (1.26, 4.27]	1.00 (0.75 ~ 1.34)	0.974	0.95 (0.69 ~ 1.29)	0.721	1.10 (0.80 ~ 1.52)	0.565
*p* for trend	0.753		0.926		0.400	
Log2-LMR						
Q1 [−0.77, 1.65]	Reference		Reference		Reference	
Q2 (1.66, 1.96]	1.19 (0.86 ~ 1.63)	0.298	1.23 (0.89 ~ 1.70)	0.219	1.10 (0.78 ~ 1.54)	0.581
Q3 (2.00, 2.37]	1.26 (0.94 ~ 1.69)	0.122	1.35 (1.00 ~ 1.82)	0.054	1.14 (0.83 ~ 1.57)	0.409
Q4 (2.38, 5.24]	1.49 (1.11 ~ 2.00)	0.009*	1.64 (1.20 ~ 2.25)	0.002*	1.27 (0.92 ~ 1.77)	0.150
*p* for trend	0.008*		0.002*		0.148	

*mean *p* < 0.05.

Model 1: Non-adjusted.

Model 2: Adjusted for race, marital status, education level, Income -to -poverty ratio (PIR), Body mass index (BMI), smoking status, and alcohol use.

Model 3: Adjusted for model 2 + infertility, pelvic inflammatory disease (PID), number of pregnancies, number of live births, age at menarche, history of diabetes, hypertension, high cholesterol levels, and cancer.

**Figure 2:**
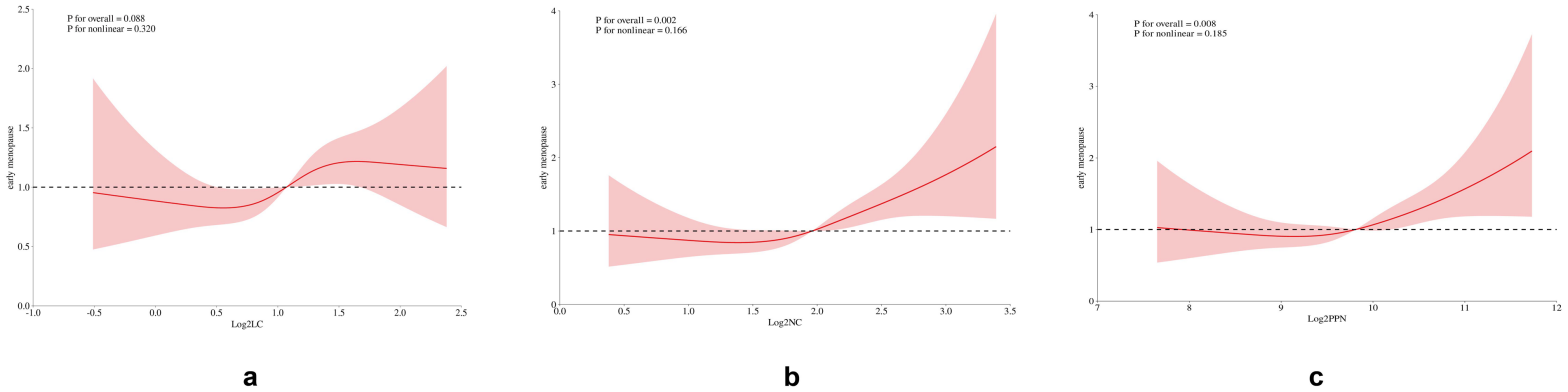
Restricted cubic spline plot of the association between inflammatory indices and early menopause. The model was adjusted for race, marital status, education level, Income-to-poverty ratio (PIR), Body mass index (BMI), smoking status, alcohol use, infertility, pelvic inflammatory disease (PID), number of pregnancies, number of live births, age at menarche, history of diabetes, hypertension, high cholesterol levels, and cancer.

A restricted cubic spline (RCS) analysis was conducted to evaluate the potential non-linearity in the association between early natural menopause and inflammatory indices (Figure 2). Notably, a linear relationship was observed between log2-LC, log2-NC, log2-PPN, and early natural menopause, with the test for non-linearity yielding a *p* value greater than 0.05.

### 3.3 Subgroup analysis

**Figure 3:**
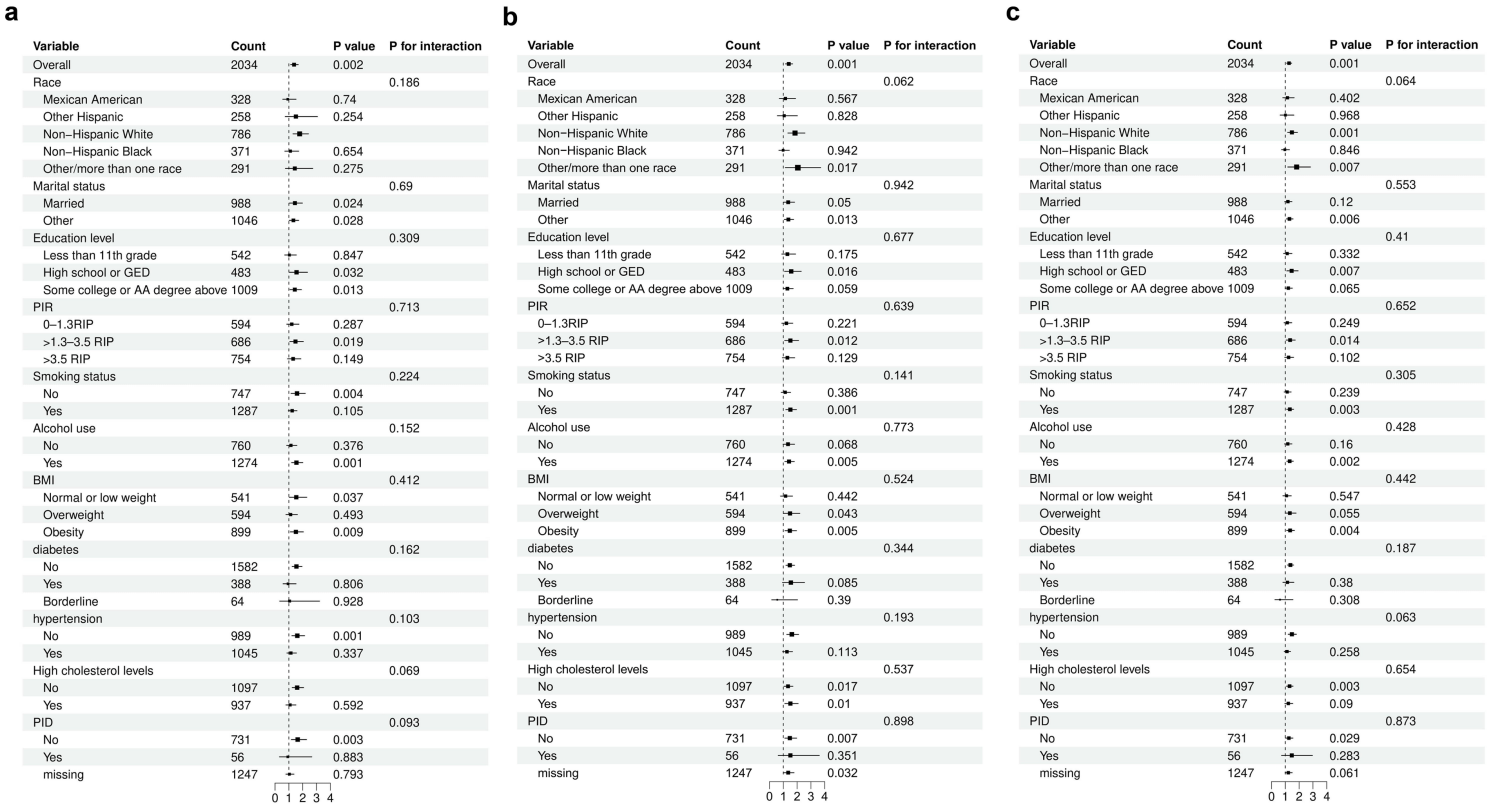
Subgroup analyses for the relationship between different inflammatory indices and early menopause. **A**: Log2-LC. **B**: Log2-NC. **C**: Log2-PPN. The model was adjusted for race, marital status, education level, Income -to -poverty ratio (PIR), Body mass index (BMI), smoking status, alcohol use, infertility, pelvic inflammatory disease (PID), number of pregnancies, number of live births, age at menarche, history of diabetes, hypertension, high cholesterol levels, and cancer.

We conducted subgroup analyses (Figure 3) to evaluate the robustness of the association between log2-LC, log2-NC, Log2-PPN and the risk of early natural menopause across various populations stratified by race, marital status, educational attainment, PIR, smoking status, alcohol consumption, BMI, and histories of diabetes, hypertension, hypercholesterolemia and PID. The association between log2-LC, log2-NC, and Log-PPN with early natural menopause was consistent across stratified populations (*P*-interaction >0.05).

## 4 Discussion

In this cross-sectional study, we investigated the relationship between various peripheral blood inflammatory indices and early menopause in a general population of women. Our findings indicated that, after adjusting for potential covariates, LC, NC, and PPN were positively associated with early menopause in American women. An increasing trend in LC, NC, and PPN corresponded with a heightened risk of early menopause. Conversely, no significant correlations were observed between the SII, PLR, NLR, and LMR with early menopause. These associations were consistent across most subgroups of participants.

Menopause is indicative of ovarian senescence. Recent studies have proposed a correlation between ovarian inflammation and diminished ovarian function, although it remains unclear whether ovarian inflammation is a causative factor or a consequence of ovarian aging. Inflammatory processes are integral to the regulation of ovarian remodeling, which is associated with follicular atresia, ovulation, and corpus luteum (CL) regression (30). Moreover, multiple prior studies conducted on mice have demonstrated that ovarian tissue experiences significant alterations during the aging process, exhibiting chronic inflammation linked to aging. This inflammation may contribute to the observed decline in oocyte quality in older adults (31–33). Early menopause is associated with a variety of etiologies, including ovarian autoimmune damage, genetic abnormalities, infectious agents, toxins, iatrogenic factors, and environmental influences (34). Nevertheless, the majority of cases remain idiopathic, with no discernible cause even after comprehensive examination. Emerging parameters from peripheral blood, including LC, NC, SII, PPN, PLR, NLR, and LMR, have been identified as markers of inflammation and immune status. Compared to many other inflammatory indices, these parameters are cost-effective, widely accessible, and routinely measured upon admission. Consequently, they have gained recognition as novel indicators associated with a range of obstetrical and gynecological conditions, such as infertility, PCOS, breast cancer, and cervical cancer. However, direct evidence establishing a connection between these novel markers and early menopause remains limited.

Several studies have investigated the relationship between peripheral blood inflammatory indices and the timing of menopause. A cross-sectional study demonstrated that early natural menopause is associated with an increase in leukocytes and neutrophils, as well as a decrease in lymphocytes, hemoglobin, and platelets, findings that align with some of our results (35). The levels of LC and NC were elevated in the early menopause group, indicating a potential association between these factors and early menopause. Natural aging of the ovary is significantly correlated with immune cell infiltration and activation of inflammation-related signaling pathways, with inflammation levels reaching a maximum during early ovarian aging (36). Different subsets of lymphocytes are implicated in the mechanisms of ovarian aging. CD8+ T lymphocytes facilitate ovulation and contribute to cell-mediated inflammation during luteal regression (37). Conventional CD4+ T cells produce cytokines, such as interleukin, TNF-*α*, and IFN-*γ*, which are essential for the ovulation process. These cytokines also promote the production of luteal prostaglandins and luteal degeneration (38). Regulatory T cells (Tregs) within the ovary serve as potent inhibitors of autoimmunity and are integral to the tolerance of allopregnancy-related tissues and autologous oocytes (39). An observed imbalance in lymphocyte counts among these women indicates a potential involvement of autoimmune mechanisms in the onset of early menopause. The specific role of neutrophils in ovarian aging remains ambiguous. However, it is hypothesized that neutrophils may contribute to both the functional decline (evidenced by reduced progesterone production) and structural deterioration (through cell death) of the corpus luteum. This process is thought to induce capillary-like formations in the endothelial cells of the corpusluteum, thereby implicating a role for corpus luteum angiogenesis (40).

Platelet and neutrophil count is an emerging biomarker, and associations between inflammatory indices and early menopause have not been documented in the literature. However, our study demonstrates a positive association between PPN and the risk of early menopause. Previous research has identified a positive correlation between PPN and female estradiol levels. Additionally, PPN is inversely associated with bone mineral density (BMD), indicating that PPN may serve as a useful predictor for the risk of osteoporosis in postmenopausal women. The divergent outcomes observed in the two studies may be attributable to the varying ages of the female participants. Consequently, further research is warranted to elucidate the relationship between PPN and early menopause in women. Moreover, previous studies have indicated that LMR is correlated with poor prognosis in patients suffering from acute ischemic stroke and advanced soft tissue sarcoma (41, 42). Previous cross-sectional studies on Chinese women with normal pregnancies have demonstrated a gradual decline in LMR levels as pregnancy progresses (43). This study is the first to identify a positive correlation between LMR levels and early menopause. However, after adjusting for potential covariates, the correlation did not reach statistical significance. Consequently, further research is warranted to elucidate the potential relationship between LMR levels and early menopause.

To the best of our knowledge, this study represents the first investigation into the association between various inflammatory indices—specifically LC, NC, SII, PPN, PLR, NLR, and LMR—and early natural menopause, utilizing data from the US National Health and Nutrition Examination Survey (NHANES). We posit that this research constitutes a crucial preliminary exploration into the relationship between inflammatory indices and early natural menopause, thereby offering substantial value for subsequent studies on the underlying mechanisms and associations. The findings underscore the clinical significance of these indices. Given that these parameters are cost-effective, straightforward to implement, and significantly correlated with the prevalence of early natural menopause, further investigation into the underlying mechanisms linking these inflammatory indices to early natural menopause is warranted.

This study has several limitations. First, the cross-sectional design precludes the establishment of causality between the risk of early natural menopause and the inflammatory indices. There may be a complex interaction between early natural menopause and the inflammatory immune response. Second, given that some information was collected through self-reported questionnaires, there is a potential for recall bias in this study. Third, the sample size was limited, and key indicators of systemic inflammation, such as C-reactive protein, were not included. Further prospective studies are required to validate these findings.

## Data Availability

Publicly available datasets were analyzed in this study. This data can be found at: NHANSE database, https://www.cdc.gov/nchs/nhanes/.

## Supplementary material

SUPPLEMENTARY FIGURE 1 Distribution of inflammatory indices among individuals included. **(A)** LC; **(B)** NC; (**C)** SII; **(D)** PPN; **(E)** PLR; **(F)** NLR; **(G)** LMR.

SUPPLEMENTARY FIGURE 2 Distribution of log2-transformed inflammatory indices among individuals included. **(A)** log2-transformed LC; **(B)** log2-transformed NC; **(C)** log2-transformed SII; **(D)** log2-transformed PPN; **(E)** log2-transformed PLR; **(F)** log2-transformed NLR; **(G)** log2-transformed LMR. SII, Systemic immune inflammation index; LC, Lymphocyte count; PPN, Product of platelet and neutrophil count; PLR, Platelet to lymphocyte ratio; NLR, Neutrophil to lymphocyte ratio; LMR, Lymphocyte to monocyte ratio.
