# Supplementary material for: Association of different inflammatory indices with risk of early natural menopause: a cross-sectional analysis of the NHANES 2013–2018
Source: Front Med (Lausanne). 2024 Nov 28;11:1490194. doi: 10.3389/fmed.2024.1490194 (PMC11638831; doi:10.3389/fmed.2024.1490194)

Supplementary Material

**Supplementary Figure 1.** Distribution of inflammatory indices among individuals included. Legend: (A)LC;(B)NC;(C)SII;(D)PPN;(E)PLR;(F)NLR;(G)LMR


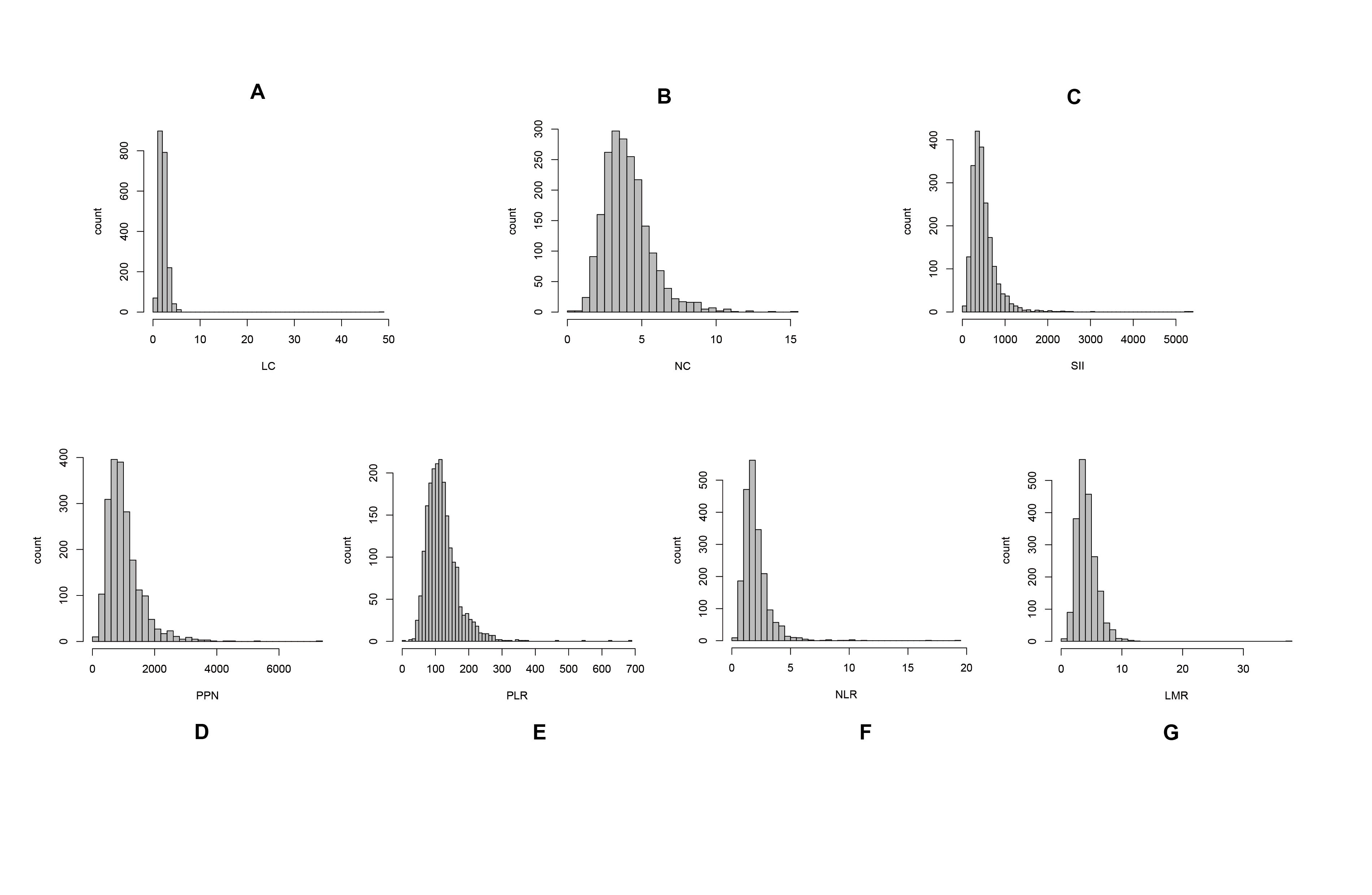


Additional file 2: Supplementary Figure 2. Distribution of log2-transformed inflammatory indices among individuals included. Legend: (A) log2-transformed LC; (B) log2-transformed NC; (C) log2-transformed SII; (D)log2-transformed PPN; (E) log2-transformed PLR; (F) log2-transformed NLR;(G) log2-transformed LMR. SII, systemic immune inflammation index; LC, lymphocyte count; PPN, product of platelet and neutrophil count; PLR, platelet to lymphocyte ratio; NLR, neutrophil to lymphocyte ratio; LMR, lymphocyte to monocyte ratio.


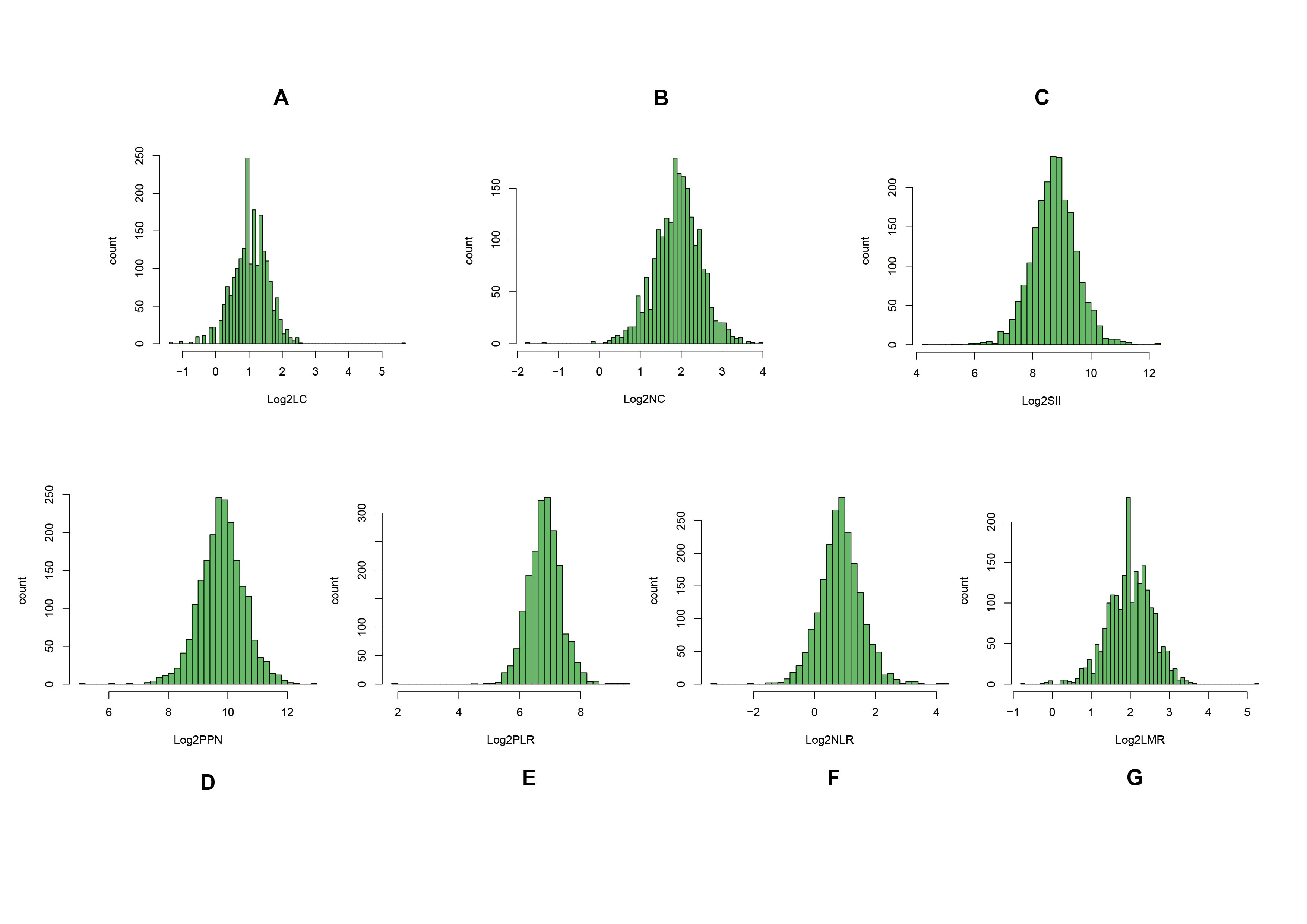

Supplement: SUPPLEMENTARY FIGURE 1 — Distribution of inflammatory indices among individuals included. (A) LC; (B) NC; (C) SII; (D) PPN; (E) PLR; (F) NLR; (G) LMR. [file Data_Sheet_1.docx]
